# “TRP inflammation” relationship in cardiovascular system

**DOI:** 10.1007/s00281-015-0536-y

**Published:** 2015-10-19

**Authors:** Tomohiro Numata, Kiriko Takahashi, Ryuji Inoue

**Affiliations:** Department of Physiology, Graduate School of Medical Sciences, Fukuoka University, Nanakuma 7-45-1, Johnan-ku Fukuoka, 814-0180 Japan

**Keywords:** TRP, Inflammation, Innate immune system, Inflammasome, Cardiovascular, Disease

## Abstract

Despite considerable advances in the research and treatment, the precise relationship between inflammation and cardiovascular (CV) disease remains incompletely understood. Therefore, understanding the immunoinflammatory processes underlying the initiation, progression, and exacerbation of many cardiovascular diseases is of prime importance. The innate immune system has an ancient origin and is well conserved across species. Its activation occurs in response to pathogens or tissue injury. Recent studies suggest that altered ionic balance, and production of noxious gaseous mediators link to immune and inflammatory responses with altered ion channel expression and function. Among plausible candidates for this are transient receptor potential (TRP) channels that function as polymodal sensors and scaffolding proteins involved in many physiological and pathological processes. In this review, we will first focus on the relevance of TRP channel to both exogenous and endogenous factors related to innate immune response and transcription factors related to sustained inflammatory status. The emerging role of inflammasome to regulate innate immunity and its possible connection to TRP channels will also be discussed. Secondly, we will discuss about the linkage of TRP channels to inflammatory CV diseases, from a viewpoint of inflammation in a general sense which is not restricted to the innate immunity. These knowledge may serve to provide new insights into the pathogenesis of various inflammatory CV diseases and their novel therapeutic strategies.

## Introduction

In the past two decades, growing attention has been directed to the pivotal roles of immunoinflammatory processes in the initiation, progression, and exacerbation of many cardiovascular diseases including atherosclerosis, post-injury vascular stenosis, myocardial infarction, heart failure, myocarditis, vasculitis, and allograft vasculopathy [1–4]. This view originates from a remarkable paradigm shift made by Ross in 1990s who first described that “excessive inflammatory-fibroproliferative response to various forms of insult to the endothelium and smooth muscle of the artery wall” is essential for the pathogenesis of atherosclerosis [5]. There is now paramount evidence that both innate and adaptive immune reactions avidly contribute to many pathological changes in the cardiovascular system, which not only involve the remodeling of the major cellular components of cardiovascular tissues but also activated immune cells migrating and accumulating therein [6]. Of particular note, solid evidence is now rapidly accumulating for the central significance of “inflammasome,” which serves as a platform mediating many innate immune reactions. The inflammasome is composed of pattern recognition receptors, apoptosis-associated speck-like protein containing a CARD (ASC) [7], and caspase-1, which by cleaving their inactive precursors generate major inflammatory cytokines interleukin (IL)-β1 and IL-18. Because of its interesting connections to both pathogen- and non-pathogen-derived cell-toxic signals such as infection, tissue damage, metabolic disorders, and other dysfunctional states of cells (see below), the activation of inflammasome is thought to be a key process leading to chronic inflammatory and autoinflammatory diseases [8]. Yet, the mechanism(s) of the activation still remains poorly understood. There are however intriguing suggestions that activation of inflammasome may depend crucially on altered ionic balance (Ca^2+^, K^+^, Cl^−^) and production of noxious gaseous mediators and its downstream mediators (ROS, NO) [9–11]. At a first glance, besides other types of channels transporting K^+^, Cl^−^, and Ca^2+^, these properties are strongly reminiscent of transient receptor potential (TRP) channels.

This review paper aims to promote our understanding about this rapidly developing field, i.e., the emerging significance of TRP channels for immunoinflammatory mechanisms in the cardiovascular (CV) system, with particular interest in innate immunity. To this end, in the first parts, we will focus on both exogenous and endogenous factors related to innate immunity and its sustained status, i.e., chronic inflammation, which possibly connect to TRP channel activities. The emerging role of autophagy to regulate innate immunity and its possible connection to TRP channels will also be discussed. In the last part, we will discuss about the linkage of TRP channels to CV inflammatory diseases, but rather from a viewpoint of inflammation in a general sense which is not restricted to the innate immunity, because the available information in this field is still greatly limited. Readers interested in another important topic, the connection of adaptive immunopathophysiology to various Ca^2+^-mobilizing mechanisms, are advised to consult with a few excellent reviews published elsewhere [12].

## Connection between TRP channels and innate immunity

The innate immune system has an ancient origin and is well conserved across species (plants, invertebrates, and vertebrates). This system defends on the frontline against microbial infection and tissue damage, consisting of environmental sensors, cellular signaling cascades, and production of antimicrobial peptides [13–16]. The recognition of pathogen-associated molecular patterns (PAMPs) or non-pathogen-associated (or danger- or damage-associated) molecular patterns (DAMPs) is the first step of activating the innate immune response, which is fulfilled via their specific receptors called the pattern recognition receptors (PRRs). The PRR family is comprised of membrane-bound Toll-like receptors (TLRs), C-type lectin receptors, retinoid acid-inducible gene (RIG)-1-like receptors, and nucleotide-binding oligomerization domain (NOD) receptor-like receptors (NLRs). TLRs have been shown to be expressed ubiquitously in cells constituting or residing in the CV system such as cardiomyocytes (TLR5), endothelial cells (TLR2/4), and macropharges/monocytes and dendritic cells (TLR1/2/4/5/8 and TLR1/2/3/7/9, respectively) [17]. Activation of PRRs by PAMPs or DAMPs is known to subsequently activate inflammasomes, whereby to promote the production of highly proinflammatory cytokines such as IL-1β and IL-18 [18]. A variety of inflammatory factors are suggested to activate the inflammasome. These include both direct and indirect signal recognition factors such as virus, bacterial toxins, particle matters, autoantibodies, and other products released from cells in dysfunction. Although there is no biochemical or morphological evidence for the coexpression with these PPRs, recent studies have suggested that TRP channels may functionally act as a cofactor PAMP/DAMP-mediated signaling for a multitude of ligands of both endogenous and exogenous origins [19]. Available evidence suggests that the connection of TLRs to TRP channels occurs through directly by LPS (TRPA1; [20]); diacylglycerol (DAG) (TRPC6; [21]); ROS (TRPM4 and TRPM7; [22], [23]); and PKC (TRPV1; [24]).

TRP channels are a family of non-selective cation channels that function as polymodal signal detectors [25, 26] and involved in a variety of body functions and diseases [27]. TRP channels are membrane proteins with six putative transmembrane segments (S1–S6) and a pore region between S5 and S6. About 30 different mammalian TRP channels have been identified and classified into six subfamilies on the basis of sequence homology: canonical or classical (TRPC; TRPC1–7), vanilloid (TRPV; TRPV1–6), melastatin (TRPM; TRPM1–8), polycystin (TRPP; TRPP2, TRPP3, TRPP5), mucolipin (TRPML; TRPML1–3), and ankyrin (TRPA; TRPA1) [28]. Different TRP channels show distinct cation selectivities and gating mechanisms and can be activated by a wide array of physical and chemical stimuli [26, 28, 29]. The regulation of TRP channels occurs at transcriptional, translational, and post-translational levels, which frequently depends on the ionic balance, microbial ligands, cytokines, or reactive oxygen species (ROS). Indeed, inflammatory transcription factors such as nuclear factor-kappa B (NF-κB), signal transducer and activator of transcription 3 (STAT3), and hypoxia-inducible factor (HIF)-1 are linked to ROS and elevated intracellular Ca^2+^ concentration. These features render TRP channels potentially effective to modulate inflammations.

In the following, we will discuss about how TRP channels are connected to respective factors and mechanisms that can activate/modulate inflammation through innate immunity.

## Factors that affect innate immune response via TRP channels (see Fig. 1)

**Fig. 1:**
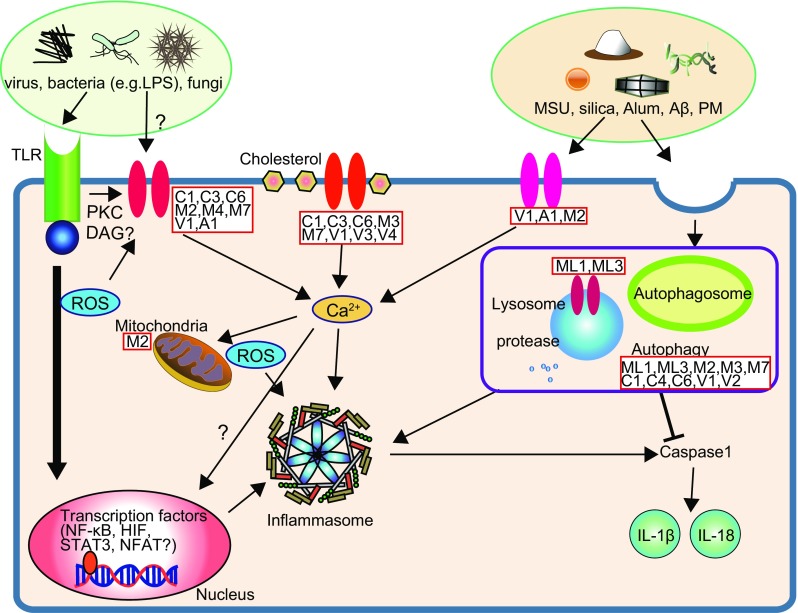
Factors affecting innate immune response via TRP channels. An incoming microorganism or virus infection can induce inflammasome activation by stimulating innate immune receptors, such as Toll-like receptors (TLRs). Alternatively, TRP channel can be activated by the constituents of microorganism (e.g., LPS) directly or indirectly through TLR activation. TRP channel is also activated by potentially toxic/harmful environmental factors, such as MSU, silica, Alum, Aβ, and PM. In addition, an essential nutrient and a cellular constituent cholesterol can modulate TRP channel activity. Abnormal TRP channel activity causes Ca^2+^ influx and thereby produces ROS. The resultant intracellular Ca^2+^ elevation and ROS may induce inflammasome activity. Moreover, dysfunction of TRP channel in mitochondria or lysosome can also activate inflammasome. It should be noted that appropriate operation of autophagy is essential to suppress the caspase-1 activity, which would prevent the production of inflammatory cytokine (IL-1β and IL-18). How TRP channel-mediated Ca^2+^ influx regulates the inflammasome activation is not fully understood; but quite conceivably, it would play a key role in innate immune response. *Question marks* denote the hypothetical pathways that will require further proof

### Viral and bacterial infections

Viral and bacterial infections are logical candidates for environmental triggers of immune reactions associated with TRP channel-dependent signaling and inflammasome activation. Upon recognition of microbial pathogens, TLRs serve as germline-encoded PRRs that play a central role in host cell recognition and responses. However, how TLR-dependent signaling links to TRP channel was unclear until very recently.

Several studies in the past few years revealed intriguing connections of TLRs to TRP channels. One study reported that hemolytic streptococcal infection affects the expression levels of at least seven TRP members, i.e., TRPC4, TRPM6, TRPM7, TRPM8, TRPV1, TRPV4, and TRPA1 [30, 31]. Another study showed that TRPC1 plays a functional role in host defense against gram-negative bacteria. Upon infection, TRPC1 (−/−) mice exhibited decreased survival, severe lung injury, and systemic bacterial dissemination. Furthermore, siRNA silencing of TRPC1 resulted in decreased Ca^2+^ entry, reduced proinflammatory cytokine production, and lowered bacterial clearance. Importantly, bacterium-mediated activation of TRPC1 was coupled with a cascade of TLR4 signaling; TLR4-dependent, TRPC1-mediated Ca^2+^ entry triggers PKCα activity to facilitate NF-κB/c-Jun N-terminal kinase (JNK) activation and augment the proinflammatory response, leading to tissue damage and eventually mortality. These findings favor the view that activation of TRPC1 is required for the host defense against bacterial infections through the TLR4-TRPC1-PKCα signaling pathway, but its excessive activity may lead to exacerbation of inflammation [32]. A similar but in-opposite-direction involvement of TRPC1-mediated Ca^2+^ entry in TLR-mediated inflammation has been demonstrated in microglia and macrophages from mice intracranially inoculated with a helminth *Mesocestoides corti* [33, 34]; it has been known that humans infected with a related helminth cestode *Taenia solium* have immunosuppressive rather than inflammatory responses in the asymptomatic phase after the infection. Experiments using soluble parasite factors from *Mesocestoides*-infected mice showed that suppression of TRPC1-mediated store-operated Ca^2+^ entry by these factors and consequent inhibition of NF-κB, JNK, and MAPK pathways are likely responsible for the immunosuppression. This novel immunosuppressive mechanism appears therapeutically useful to prevent the initiation of TLR-dependent inflammatory response via suppression of TRPC1 activity. In cultured macrophages, however, degradation of TRPC1 by caspase-11, an inducible caspase which is activated by NLRP3 inflammasome activator lipopolysaccharide (LPS), was found to increase the secretion of IL-1β. This negative regulation by TRPC1 occurred independently of caspase-1 cleavage or cell death [35] and thus likely reflects a distinctive mechanism from those described above. Consistently, a higher IL-1β secretion was observed in the sepsis model of TRPC1-deficient mice made by intraperitoneal LPS injection [35]. Although there is always a caveat to the relevance of knockout studies such as compensatory expression of homologous or other types of molecules which might affect downstream signaling (similar arguments may also hold for other TRP knockout models; see below), these results collectively imply the presence of multiple signaling pathways involving TRPC1 that regulate TLR-mediated inflammation. Further detailed analyses will be necessary to understand how manipulation of TRPC1 activity could be utilized for immune-modulatory interventions of inflammation.

In addition, there is evidence linking other TRP members to TLR-mediated signaling. In airway smooth muscle (ASM) cells, exposure to a proinflammatory cytokine TNF alpha (TNFα) or a mixture of allergens (ovalbumin, house dust mite, *Alternaria*, and *Aspergillus* extracts) causes both acute and chronic inflammations. These inflammatory responses involve at least in part increased secretion of brain-derived neurotrophic factor (BDNF) in a manner dependent on TRPC3-mediated Ca^2+^ entry [36]. In endothelial cells (ECs), endotoxin (LPS) induces pathological vascular leakage. This occurs through the interaction between TLR4 signaling and TRPC6-mediated Ca^2+^ entry, which causes increased endothelial permeability via activation of non-muscle myosin light chain kinase (MYLK) and NF-κB. Genetic deletion of TRPC6 rendered mice resistant to endotoxin-induced barrier dysfunction and inflammation and protected against sepsis-induced lethality [21].

TRPM4 is causally related to LPS-induced endothelial cell death via intracellular Na^+^ overloading. Pharmacological inhibition of TRPM4 activity with 9-phenathrol or glibenclamide was found to attenuate this consequence, suggesting a therapeutic potential of TRPM4 for endotoxin shock [22]. TRPM7-mediated intracellular concentration of Ca^2+^ ([Ca^2+^]_i_) elevation serves as a key regulator for endotoxin-induced endothelial fibrosis through endothelial to mesenchymal transition [23]. This channel is also implicated in LPS-induced endothelial cell migration via TLR4/NF-κB pathway [37]. TRPM2-deficient mice shows compromised innate immunity against *Listeria monocytogenes* infection which allows uncontrolled replication of the bacteria with significantly reduced production of IL-12 and interferon-γ [38]. Consistent with this finding, in a cecal ligation and puncture (CLP)-induced mouse sepsis model, genetic disruption of TRPM2 was found to cause impaired host defense, leading to increased mortality associated with increased bacterial burden, organ injury, and systemic inflammation. Interestingly, this finding appears to reflect failed upregulation of heme oxgenase (HO)-1 in macrophages which is normally induced by TRPM2-mediated Ca^2+^ influx and essential for bacterial clearance [39].

In recent years, the potential benefits of TRPV1 activation have been recognized for the abatement of inflammatory response. For example, in *Helicobacter pylori*-positive patients, the genetic polymorphism of TRPV1 945G>C has been suggested to be one of the pathophysiological factors of functional dyspepsia [40]. In murine sepsis models, genetic or pharmacologic disruption of TRPV1 can affect mortality, blood bacteria clearance, and cytokine response, in such a pattern that may vary according to the sepsis-inducing events and the methods of TRPV1 disruption [41, 42]. In salivary glands, polyinosinic-polycytidylic acid or LPS activates, via TLR4 activation, NF-κB by IκB-α degradation and phosphorylation to release highly proinflammatory cytokines TNFα and IL-6. Capsaicin inhibits this process by interacting with the NF-κB pathway whereby to potentially alleviate inflammation of salivary glands [43]. Indeed, in healthy human subjects as well as patients, capsaicin has been suggested to have a therapeutic potential alone or in combination with other non-steroidal anti-inflammatory drugs [44], and in a mouse CLP model, capsaicin is shown to relieve the damaging impact of sepsis through TRPV1 activation [44, 45]

### MSU, cholesterol, amyloid-β, and ambient particle matter

#### MSU

A uric acid crystal, monosodium urate (MSU), has emerged as an important factor for both gouty arthritis and immune regulation. This simple crystalline structure appears to activate innate host defense mechanisms in multiple ways and trigger robust inflammation and immune reactions. When MSU enters the cell, MSU further triggers NLRP3 inflammasome activation, but the activation mechanism responsible is still elusive. A number of reports suggested that sensory TRP channels such as TRPV1 and TRPA1 may contribute to this activation process [46, 47]. However, this interesting hypothetical link relies on the intracellular Ca^2+^ measurements, and whether MSU crystals can directly activate TRPV1 [46] or TRPA1 [47] has not been shown. Considering that MSU crystal acts as an oxidative stress to facilitate ROS production, it may activate TRPA1 and TRPV1 via ROS production. Nevertheless, the fact that TRPV1 and TRPA1 mediate MSU crystal-induced inflammation and pain in experimental models strongly supports their undoubted roles as inflammatory mediators [46–50].

#### Cholesterol

A high serum level cholesterol (hypercholesterolemia), a risk factor for CV disease, promotes inflammatory responses including TLR signaling, inflammasome activation, and the production of monocytes and neutrophils in bone marrow and spleen [51]. Cholesterol was also shown to have a significant impact on several different types of TRP channel activities [52]. Cellular cholesterol affects arterial reactivity to endothelin-1 (ET-1). In endothelium-denuded caudal artery, cholesterol depletion by methyl-β-cyclodextrin (mβcd) treatment attenuated vasoconstriction to ET-1, with paralleled reduction of store-operated Ca^2+^ entry via TRPC1 [53]. Similarly, cholesterol-mediated activation was observed for other TRP channels. In TRPC3 expressing HEK cells, application of cholesterol enhanced TRPC3 activity [54]. In prostate cancer cells which endogenously express TRPM7, cholesterol-mediated activation of TRPM7 is important for initiation and/or progression of the cancer [55]

In vascular smooth muscle cells (VSMCs), TRPM3 expression was detected at both mRNA and protein levels. In freshly isolated aorta, constitutively active TRPM3 channel positively modulated the contractile responses independently of L-type calcium channels; elevation of cholesterol suppressed TRPM3 channel activity [56].

These results collectively suggest that an appropriate level of cholesterol may be requisite for normal contractility of arteries, and its excess level may lead to CV diseases as well as makeup of microenvironments propensitive for cancer development.

#### Amyloid-β

The fibrillar peptide amyloid-β (Aβ) is a main pathogenic factor of Alzheimer’s disease (AD). It has been reported that activation of TRPM2 contributes to Aβ- and oxidative stress-induced striatal cell death in rat striatum [57]. The activation of TRPM2 is thought to occur through direct and indirect pathways. Accumulation of Aβ which increases vascular oxidative stress via mitochondrial dysfunction results in sequential occurrences of DNA damages, poly-ADP-ribose polymerase activation, and ADP-ribose production in ECs, which leads to their dysfunction via TRPM2-mediated intracellular Ca^2+^ overload [58]. The resultant cerebrovascular dysfunction may accelerate the AD pathogenesis by reducing the cerebral blood and glucose supply, increasing susceptibility to vascular insufficiency, and further promoting Aβ accumulation [59]. The other oxidative stress-sensitive TRP channels such as TRPV1, TRPV4, and TRPC1 are also implicated in Aβ-induced damages of other cell types including glia [60–63], smooth muscle cells [64], and ECs [65].

#### Ambient particle matter

Exposure to ambient particulate matters (PMs) is a significant risk factor to increase respiratory and cardiopulmonary morbidity and mortality, but the mechanism underlying remains largely unknown. In addition to the implications of sensory nerve TRPV1 and TRPA1 in airway hypersensitivity and inflammation which involve environmental noxious stimuli [66], there is evidence that PM acts as a proinflammatory agent to the endothelium and increases vascular permeability in vitro and in vivo via ROS generation. This suggests the possibility that oxidative stress-sensitive TRP channels may be involved in PM-mediated pathophysiology. Indeed, TRPM2, TRPV1, TRPV4, and TRPA1 have been implicated in cellular and tissue damages caused by PM-mediated oxidative stresses associated with diesel exhaust, wood smoke, and other concentrated ambient particles [67–75].

### Autophagy and lysosomal function

Autophagy is a highly evolutionarily conserved catabolic process to degrade and recycle cytoplasmic contents via a lysosomal route for reuse in downstream metabolism. It becomes increasingly clear that insufficiency of autophagy is an important pathogenic mechanism for inflammatory diseases [76]. Recently, a possible link between autophagy deficiency and increased inflammasome activation was suggested. The mechanism proposed includes the following causal sequence of cellular events: inefficient mitophagy, accumulation of damaged mitochondria, increased ROS production, and ROS-mediated inflammasome activation which occurs either directly or indirectly via DNA damage and secondary inflammatory signaling(s). Autophagy deficiency also reduces the efficiency of lysosomal degradation and thereby facilitates the accumulation of intra-lysosomal lipids and cholesterol crystals. This then leads to lysosomal membrane destabilization, lysosomal leakage, and inflammasome activation [77]. A similar consequence of autophagy deficiency can be expected for inefficient lysosomal degradation of damaged organelles and proteins. Therefore, normal function of the autophagy system is indispensable for keeping the cell healthy.

TRPML1 is an important player in endosomal sorting and transporting processes at the late endocytotic phase, specifically the formation of late endosome-lysosome hybrid vesicles [78–81]. In other words, the role of this channel is to control the delivery of cellular materials to lysosomes, an essential process of autophagy [82–84]. Altered activity of TRPML1 has been implicated in lysosomal dysfunction and impaired autophagy associated with AD-linked presenilin-1 mutations. In this pathological state, disrupted lysosomal acidification due to defective vesicular ATPase activity are thought to be primarily responsible for lysosomal and autophagy deficits, but concurrently, abnormal cytosolic Ca^2+^ elevation occurs via facilitated Ca^2+^ efflux through TRPML1 channel. However, correcting this abnormal Ca^2+^ homeostasis alone is not sufficient to restore normal lysosomal proteolytic and autophagic activities, thus suggesting that TRPML1 may play a permissive role in this process [85]. In this regard, it may deserve to mention that in humans, mutations in the gene encoding TRPML1 channel (*MCOLN1*) are the cause of the neurodegenerative disorder mucolipidosis type IV (MLIV) [86].

TRPML3 is a novel Ca^2+^ channel that plays a crucial role in the regulation of cargo trafficking along the endosomal [87, 88] and autophagosomal maturation [89] pathways. In infected bladder epithelial cells (BECs), TRPML3 triggers a non-lytic expulsion of bacteria (which is a powerful cell-autonomous defense strategy) to rapidly reduce infectious burden. This lysosomal channel is capable of sensing uropathogenic *Escherichia coli*-mediated lysosome neutralization and, in turn, releasing Ca^2+^, thereby triggering lysosomal exocytosis to expel the bacteria.[90]

The full-length form of TRPM2 channel (TRPM2-L) has a short splice variant consisting of only the N terminus and the first two transmembrane segments and lacking a pore domain (TRPM2-S). In expression system, coexpression of TRPM2-S suppressed oxidant-induced Ca^2+^ entry through TRPM2-L and subsequent cell death, presumably through a negative physical interaction [91]. Although its pathophysiological significance had been unclear, a recent study has revealed an interesting connection of this short variant (TRPM2-S) to autophagy. Mitochondrial homeostasis is dynamically regulated by the processes of autophagy/mitophagy and mitochondrial biogenesis. As compared with tumor cells expressing TRPM2-L isoform abundantly, those expressing TRPM2-S showed the accumulation of damaged mitochondrial DNAs with increased levels of unremoved heat shock protein 60 (Hsp60) and a mitochondrial protein translocase of outer membrane 20 (Tom20) in mitochondria. These results are interpreted to suggest that oxidant-induced Ca^2+^ entry mediated by TRPM2 may be crucial to maintain normal autophagy/mitophagy activity [92].

Oxidative stress induces pleiotropic responses ranging from cell survival to death. A recent study has given an interesting explanation about these differential cell fates, i.e., involvement of distinct poly(ADP-ribose) polymerase (PARP) isoforms (PARP1, PARP2) and distinctive cellular localization of TRPM2 channel. PARPs are enzymes producing poly(ADP-ribose) and, in conjunction with poly(ADP-ribose) glyocohydorase, capable of activating TRPM2 channel via immediate conversion of poly(ADP-ribose) into monomeric ADP-ribose. Under moderate oxidative stress conditions (5 mM H_2_O_2_), plasma membrane TRPM2 is under the control of PARP1, activation of which leads to the phosphorylation of p38, SAPK/JNK, and cAMP response element-binding protein (CREB)/ATF-1. This ultimately induces autophagy, thereby allowing cell survival. In contrast, high oxidative stress (10 mM H_2_O_2_) triggers late autophagy steps and PARP2 activation, leading to cell death with the activation of lysosomal TRPM2 channel [93].

## TRP channels may link to persistent activation of inflammasome (see Fig. 2)

**Fig. 2:**
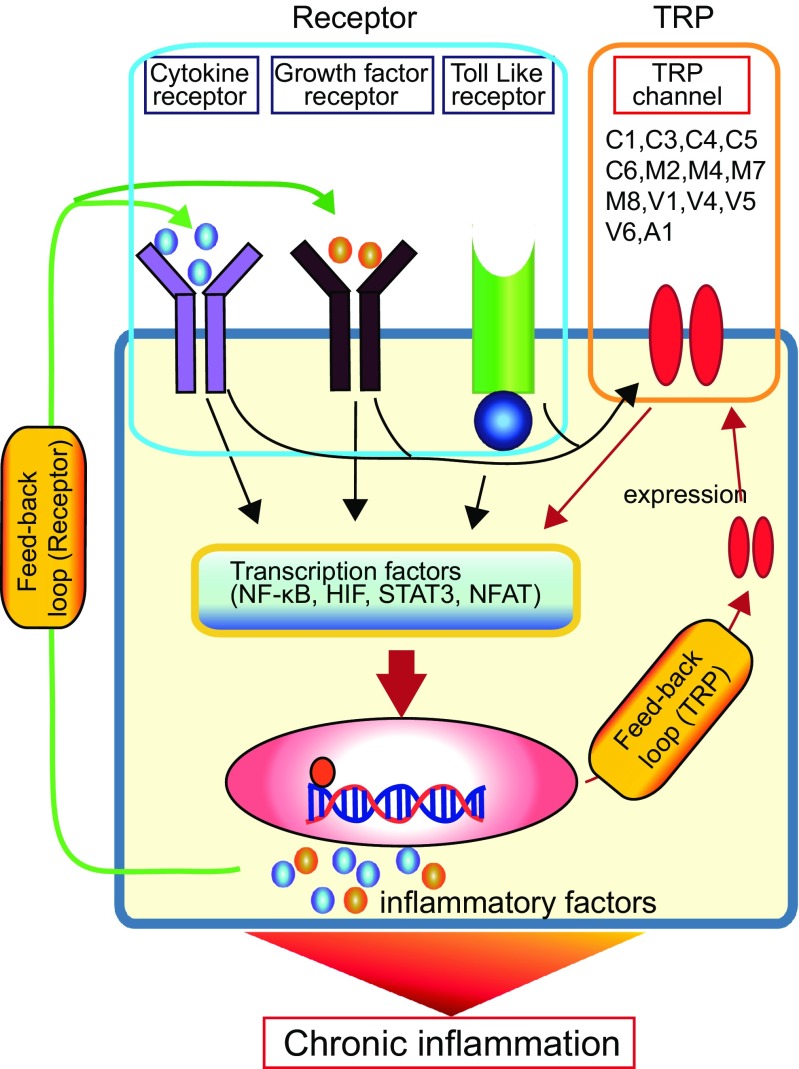
Possible link of TRP channels to persistent inflammasome activation. Inflammation are mediated by signal transduction from both inflammation-related receptors and stress-sensing TRP channels to transcription factors. The “*Receptor*” pathway is activated via alteration of internal or external environmental factors in affected cells, including abnormal upregulation or persistent activation of receptors (e.g., cytokine receptors, growth factor receptors, Toll-like receptors) with increased kinase activity, excessive ROS production, and intracellular Ca^2+^ perturbation. The “*TRP*” pathway can also be activated by alteration of internal or external environmental factors and is susceptible to the regulations at functional and expression levels. Importantly, both pathways may intersect to modulate each other, since many of the environmental factors associated with inflammation are physical and chemical cell-stressing stimuli. Besides being involved in proliferation, survival, migration, and differentiation, transcription factors can induce the expression of many proinflammatory cytokines and other inflammatory mediators. Importantly, the receptors for many of these cytokines, chemokines, and mediators can further activate the inflammatory transcription factors, thereby forming autocrine and paracrine feedback loops. This would then result in the continuous amplification and promotion of inflammatory reactions leading to chronic inflammation

The early inflammatory response is absolutely essential for the elimination of pathogens, but the termination of the process is equally an important step. Failure to control inflammation leads to immunopathology, including CV diseases and organ dysfunctions. Over time, this leads to fibrotic or cancerous transformation or chronic inflammation.

There are three aspects in the progression of inflammation with respect to inflammasome activity. The first is the “priming” signal that enhances the gene expression of inflammasome components. The second is the “activation” signal that promotes the assembly of inflammasome components. And the last is the “amplification” signal that drives a feedback signal amplification loop. The NLRP3 inflammasome activates caspase-1, which then promotes maturation and secretion of two potent inflammatory cytokines: IL-1β and IL-18. The NLRP3 inflammasome responds not only to pathogens but also to a variety of “danger signals” released in inflamed tissues including cytokines, tissue degradation products, etc. This leads to the formation of a dangerous positive feedback loop which continuously exacerbates the NLRP3 inflammasome response. Under these pathological conditions, the activation of transcription factors, e.g., NF-κB, nuclear factor of activated T cell (NFAT), STAT3, and HIF-1, by inflammatory cytokines appears crucial for the amplification loop of inflammasomes [94–97]. Therefore, the suppression of this amplification loop is of key importance to effectively eliminate infection by stopping acute inflammation and/or restoring homeostatic regulation.

Concentrations of both extracellular and intracellular Ca^2+^ can increase at the sites of infection, inflammation, or immune cell activation. It has been shown that increased extracellular calcium could act as a danger signal and an amplifier of inflammation via activation of the G protein-coupled calcium sensing receptor—phosphatidylinositol/Ca^2+^—NLRP3 inflammasome signaling pathway [98]. Therefore, Ca^2+^ influx may serve as an important control point for inflammasome activity that needs to be tightly regulated by the host in order to avoid an excessive production of cytokines or overt cell death. Moreover, proinflammatory transcription factors, in particular, Ca^2+^-dependent ones, may play central roles in this control. In this section, we attempt to summarize the connection between inflammatory transcription factors and TRP channels.

### NF-κB

NF-κB consists of a family of transcription factors that play critical roles in inflammation, immunity, cell proliferation, differentiation, and survival [99]. One of the factors known to activate NF-κB is an elevation in [Ca^2+^]_i_ [100, 101]. There are several papers linking Ca^2+^-permeable TRP channels to NF-κB-mediated inflammatory reactions; suppression of TRPC1-mediated Ca^2+^ entry inhibited NF-κB activation, which is associated with immunosuppressive mechanism in helminth infections [34]; pharmacological inhibition of TRPM7 channel suggested its involvement in LPS-induced EC migration via the TLR-NF-κB signaling [37]; endotoxin-induced lung injury involves TLR4-mediated NF-κB activation in a manner dependent on TRPC6-mediated Ca^2+^ entry [21].

Therapeutic potential of targeting NF-κB has been validated by its decoy treatments in a number of inflammatory CV diseases, including myocarditis, post-stenting coronary restenosis, and coronary hypertophic proliferation [102–104].

### STAT3

The STAT3 has a dual role: both transducing signals through the cytoplasm and functioning as a transcription factor in the nucleus. STAT3 can be activated by extrinsic pathways, i.e., environmental factors, such as ultraviolet (UV) radiation, chemical carcinogens, infection, stress, and cigarette smoke, through growth factor, cytokine, Toll-like, adrenergic, and nicotinic receptors, respectively [95]. Persistently activated STAT3 mediates cell proliferation, survival, and invasion during inflammasome activation. Several studies suggest that TRP channels may regulate STAT3 activity.

Receptor-induced phosphorylation of cellular Janus kinase 2 (JAK2) or c-Jun and STAT3 are regulated by TRPC1- and TRPC6-mediated Ca^2+^ influxes [105–108]. This could cause persistent inflammation, resulting in chronic inflammation with global tissue changes and injury. The Ca^2+^ transporting activity of TRPM7 is closely associated with the activation of the JAK2/STAT3 and/or Notch signaling pathways, which, in turn, induces ischemic neuronal cell death, metastatic transformation of breast cancer cells or proliferation, migration and invasion of glioma stem cells, and the fingerprints of sustained or chronic inflammation [109–111]. Albeit little evidence, it is tempting to speculate that constitutive Ca^2+^ permeating activity TRPM7 channel might effectively drive, via Ca^2+^-dependent activation of STAT3, a feedback cycle of persistent activation of inflammasome toward chronic inflammation. A different line of evidence suggests that TRPV1 activates the STAT3 and NF-κB signaling pathways and thereby facilitates the expression of anti-inflammatory neuropeptides [112]. This hints a unique protective role of TRPV1 against inflammation, which is rarely seen for the other types of TRP channels.

### HIF-1

HIFs are transcription factors that respond to changes in available oxygen in the cellular environment. However, a recent research suggests that, in certain pathological settings, HIF induction in normoxia likely causes serious consequences encompassing chronic inflammatory components. Chronic inflammation can be a self-perpetuating process so that it may continuously distort cellular microenvironments as the result of aberrantly active transcription factors. Consequent alterations in growth factors, chemokines, cytokines, and redox balance occur within the cellular milieu that, in turn, provide the axis of growth and survival needed for de novo development of cancer and metastasis [113]. In addition, a recent study suggested that cAMP/PKA/CREB/HIF-1α pathway is important for sustained inflammasome activity [114]. Considering that TRP channels serve as important Ca^2+^ entry routes associated with cellular stresses, it is possible that abnormal TRP channel activity would damage the intracellular microorganelles and disturb the redox balance. In fact, there are many reports found for the linkage between HIF-1 and TRP channels (e.g., TRPC1 [115–117], TRPC3 [118], TRPC5 [119], TRPC6 [120, 121], TRPM2 [92, 122], and TRPM7 [123]).

### NFAT

The calcineurin-NFAT signaling plays a pivotal role in the transcription of cytokine genes and other genes critical for the T cell-mediated adaptive immune responses [124]. However, later studies identified its more ubiquitous roles in other organ systems [125]. For example, an established role of NFAT in the CV system is a hub mediator of hypertrophic signaling. In the heart, both mechanical and neurohormonal stresses activate the calcineurin-NFAT signaling to induce prominent pathological hypertrophy. The members of TRPC subfamily have been implicated in the activation step of this signaling as the stress-sensing Ca^2+^-permeating channels [126–128]. In blood vessels, therapeutic benefits of an immunosuppressant cyclosporin A is known for transplant vasculopathy, part of which is causally related to the inhibition of calcineurin-NFAT signaling in ECs [129]. In diabetic mice, pharmacological inhibition of NFAT (by A-285222) reduced atherosclerotic lesion by inhibiting cytokine release and adhesion molecule expression [130]. Albeit scanty information, involvement of TRPC members has been suggested for inflammatory responses in renal podocyte injury and structural and functional remodeling after myocardial infarction via activation of the calcineurin-NFAT signaling [131, 132]. There is however little evidence yet obtained that directly links TRP channels to chronic inflammatory diseases (e.g., atherosclerosis) through this signaling pathway.

## TRP inflammation relationship in CV diseases

Many CV diseases are tightly associated with inflammatory/immune responses in their pathogenesis. This include a wide range of vascular diseases (endotoxin shock, acute vasculitis, atherosclerosis, post-operative stenosis, allograft vasculopathy), cardiac diseases (cardiac hypertrophy, dilated cardiomyopathy, myocardial infarction or ischemia/reperfusion injury, and myocarditis) [133–142], and several autoimmune diseases in the CV system [8]. In the final section of this review, we will briefly overview what is presently known about some of these CV diseases in terms of TRP channel physiology and pathophysiology (see also Table 1).

**Table 1:** Therapeutic potential of TRP channel in cardiovascular disease

Disease	TRP	The potential benefit for disease (+): positive regulation is good, (−): negative regulation is good	Ref.
Atherosclerosis	C1	C1(+): Vascular contractility in cholesterol depletion C1(−): Coronary artery disease in metabolic syndrome	[53, 143, 144]
	C3	C3(−): The size of atherosclerotic lesions	[145, 146]
		C3(−): Adhesion of monocyte in coronary artery ECs	
	C6	C6(−): Migration and apoptosis of EC in atherosclerotic arteries (by microRNA-26a)	[147, 148]
	C6, V1	C6(−): V1(−): Lysophosphatidylcholine-induced infiltration of monocyte	[149]
	V1	V1(+): Evodiamine-induced angiogenesis and atherosclerosis V1(+): ox-LDL-induced foam cell formation by inducing autophagy in vascular SMC V1(+): ox-LDL-induced lipid accumulation and TNFα-induced inflammation in macrophages V1(+): Adhesion monocyte and EC	[150–153]
Neointimal hyperplasia	C1	C1(−): Remodeling of SM	[154]
	M2	M2(−): Remodeling of SM	[155]
Hemorrhagic Shock	C1/C4	C1/C4(−): Development of vasospasm after subarachnoid hemorrhage	[156]
	V1	V1(−): Survival rates in hemorrhagic shock model	[157]
Cardiovasculitis		ND	
Cardiac hypertrophy, dilated cardiomyopathy	C1	C1(−): Related to cardiac fibrosis in Duchenne muscular dystrophy model mice C1(−): Increasing endothelin-1 vasoconstrictor reactivity through Ca^2+^/ROS/NFATc3 C1(−): Maladaptive cardiac hypertrophy and failure thorough Ca^2+^/calcineurin/NFAT pathway C1(−): Development of cardiac hypertrophy through Ca^2+^/calcineurin/NFAT pathway in hypertension in sleep apnea	[158–161]
	C3	C3(−): Ca^2+^-dependent production of CaMK II and ROS in dilated cardiomyopathy C3(−): Cardiac hypertrophy via GATA4 and TRPC3 (antagonist: miR-26b) C3(−): Cardiac hypertrophy via a positive feedback mechanism through Ca^2+^/calcineurin/NFAT signaling	[162–164]
	C3/C6	C3/C6(−): Hypoxia-induced HIF1α, leading to expression, enhanced Ca^2+^/calcineurin signals C3/C6(−): Ang II-induced cardiac hypertrophy through Ca^2+^/calcineurin/NFAT signaling	[118, 165]
	C6	C6(−): Ca^2+^/calcineurin/NFAT regulatory loop that drives pathologic cardiac remodeling C6(−): Ang II-induced cardiac hypertrophy C6(−): Development of cardiac hypertrophy through GATA4 and NFATc4	[166–168]
	V1	V1(+): Long-term high-salt diet-induced cardiac hypertrophy and fibrosis V1(+): Pressure overload-induced cardiac hypertrophy and fibrosis	[169, 170]
	V2	V2(−): Ventricular dilation and fibrosis through CaMK II and ROS in DCM patients and three DCM model mice	[171]
	M4	M4(+): Hyperplasia in the cardiac hypertrophy M4(+): Ang II-induced cardiac hypertrophy through Ca^2+^/calcineurin/NFAT pathway	[172, 173]
Allograft		ND	
Coronary, myocardial infarction	C3/C4/C6	C3/C4/C6(−): After MI induce Ca^2+^/calcineurin/NFAT pathway pathway, activate cardiac hypertrophy, reduces contractility reserve C3/C6(+): MI-induced injury and cardiomyocyte apoptosis are alleviated by BDNF/TrkB axis	[132, 174]
	M4	M4(−): MI cause cell death and decrease β-adrenergic cardiac reserve	[175]
	V1 V1	V1(+): Post-MI enhances fibrosis and impairs myocardial contractile performance V1(+): Acute myocardial ischemia augments the Bezold–Jarisch reflex	[176, 177]
	V2	V2(+): M1 macrophage infiltration after MI	[178]
Ischemia reperfusion	V1	V1(+): Myocardial I/R injury can be protected by 12-lipoxygenase-derived eicosanoids V1(+): PAR2-induced cardiac protection against I/R injury V1(+): Cardiac performance in I/R-injured diabetic heart V1(+): Acute MI	[179–182]
	M2	M2(−): Myocardial I/R injury in neutrophil M2(−): I/R induce TNFα, caspase-8 activation, ROS production, PARP-1 activation, ADP-ribose production, that contribute to cardiomyocyte cell death	[183, 184]
	M4	M4(−): I/R injury	[185]
Stenosis, systemic lupus erythematosus	C5/C6	C5/C6(−): Linkage analysis data for infantile hypertrophic pyloric stenosis	[186]
Autoimmune, autoantibody		ND	
Hypertension, diabetes		ND	

*EC* endothelial cell, *SMC* smooth muscle cell, *ox-LDL* oxidized low-density lipoprotein, *TNFα* tumor necrosis factor, *I/R* ischemia reperfusion, *ND* no data, *Ang II* angiotensin II, *miR* microRNA, *ADP* adenosine diphosphate, *PARP-1* poly[ADP-ribose] polymerase-1, *DCM* dilated cardiomyopathy, *ROS* reactive oxygen species, *MI* myocardial infarction, *BDNF* brain-derived neurotrophic factor, *TrkB* tropomyosin receptor kinase B, *PAR2* protease-activated receptor-2, *NFAT* nuclear factor of activated T cells

### Atherosclerosis

Atherosclerosis is a progressive inflammatory disease that disintegrates the structure and function of blood vessels through lipid deposition and activation of innate/adaptive immune reactions. There are good evidence to suggest that TRP channels are involved in the respective stages of atherosclerosis. This will be described below, in a stage-by-stage manner.

The main initiation factor for atherosclerosis is the oxidized low-density lipoprotein (ox-LDL), which affects the endothelial surface and thereby induces EC dysfunction. But, other inflammatory components/elements (virus, bacteria, toxins, toxic nutrients and metabolites, ambient particles, autoantibody, heat shock protein [187]; see also above) also promote atherosclerosis. Among TRP channels expressed in the CVS, two reports suggest proatherogenic potential of TRPC6 channel. It is reported that a cardiovascular risk factor, lysophosphatidylcholine (LPC), facilitates TRPC6 translocation to cell membrane causing a rapid Ca^2+^ influx in EC. This, in turn, induces the externalization of TRPC5, thereby allowing sustained Ca^2+^ influx through TRPC6/TRPC5 complex. This cascaded TRPC6/TRPC5 activation causes the inhibition of EC migration which is essential for the healing of atherosclerotic arteries [147]. Endothelial expression of microRNA miR-26a is reduced in the aortic intima of atherogenic ApoE (−/−) mice and ox-LDL-treated human ECs, whereas overexpression of miR-26a induces EC apoptosis. miR-26a acts as a negative regulator of TRPC6, activation of which allows Ca^2+^ influx activating the mitochondrial apoptotic pathway associated with atherosclerosis. Thus, abnormal TRPC6 activity can induce apoptotic EC death, and application of miR-26a may be able to reduce the atherosclerotic lesion [148]. These results provide, albeit indirect, evidence to support non-trivial roles of TRPC6 in the progression of atherosclerosis.

At the site of injury, ECs allow the entry of monocytes and lymphocytes into the vessel wall. In the early stage of this process, the adhesion of monocytes to the endothelial surface is supported by increased intracellular Ca^2+^ concentration in ECs. When the monocyte contacts EC, Ca^2+^ influx into EC is activated, which further strengthens the adherence of monocytes. Pharmacological inhibition of TRPV1 reduces the number of adherent monocytes, so that activation of TRPV1-mediated Ca^2+^ influx likely enables strong adhesion of monocytes to EC. In a similar context, TRPC3-mediated Ca^2+^ influx has been implicated in ATP-induced expression of vascular cell adhesion molecule-1 (VCAM-1) which is critical to recruit monocytes to EC [145, 150].

After injury, monocytes transmigrate across the damaged endothelium and enter the intimal layer of the vessel wall. Thus, reducing monocyte infiltration is thought to be one of the powerful strategies to attenuate the progression of atherosclerosis. A major atherogenic agent LPC can induce a strong chemotaxis of monocytes which appears to require Ca^2+^ influx. Pharmacological characterization of LPC-activated Ca^2+^-permeable currents strongly suggested that activation of both TRPC and TRPV1 is necessary for the optimal chemotaxic activity [149]. In this regard, these two TRP channels could be good molecular targets for anti-atherosclerotic therapy.

The monocytes that have migrated into the intima differentiate into macrophages and uptake ox-LDL. These lipid-laden macrophages are known as foam cells. Although the main foam cells are derived from macrophages, VSMCs can also transform to foam cells. Interestingly, activation of TRPV1 by capsaicin impedes the transformation of ox-LDL-treated VSMCs to foam cells by rescuing otherwise impaired autophagy by ox-LDL [151]. Moreover, TRPV1 activation protected macrophages from ox-LDL-induced lipid accumulation and TNFα-induced inflammation. These results indicate that activation of TRPV1 by capsaicin is another effective strategy to inhibit atherosclerosis.

After transformation, foam cells start to produce inflammatory cytokines. The released inflammatory cytokines trigger further transformation of VSMCs which gain the ability to migrate from the medial to intimal layer. These inflammatory responses make atherosclerotic plaques through necrosis and apoptosis, completing the clinical picture of atherosclerosis. In cultured RAW264 macrophages, LPS-induced production of cytokines (TNFα, IL-6) was shown to depend on TRPV2-mediated Ca^2+^ influx. Thus, in this late stage of atherosclerosis, targeting TRPV2 may be another novel strategy to suppress inflammatory cytokine production and thus formation of atherosclerotic lesions [188].

### Vascular stenosis after bypass surgery and angioplasty

Bypass surgery and angioplasty with stenting often causes the vascular injury. Injured VSMCs then undergo transformation from quiescent and contractile to invasive and proliferative states. Such phenotype switching is an important process to recover vascular contractility but can also be part of the cause of occlusive vascular diseases including atherosclerosis and adverse responses accompanying neointimal hyperplasia. Several lines of evidence suggest that excessive (constitutively active) TRPC1 and (ROS-induced) TRPM2 activities in VSMCs are involved in the neointimal hyperplasia induced after vascular cuff injury in rat and mouse models, respectively. These changes were accompanied by enhanced cell cycle activity and inhibited by specific antibodies raised against the third extracellular loop of TRPC1 or TRPM2 channel (E3 antibodies). Similar increased expression of TRPC1 was also observed in the intimal layer of human vein graft samples. Thus, these channels may be novel therapeutic targets for occlusive vascular diseases [154, 155].

### Cardiac hypertrophy, dilated cardiomyopathy

In cardiomyocytes, Ca^2+^ transients convey information to both contraction and gene transcription. Changes of cytoplasmic Ca^2+^ in cardiomyocytes are controlled by a variety of ion channels, the Na^+^/Ca^2+^ exchanger, Ca^2+^ pumps, and Ca^2+^-binding proteins. Several recent studies using animal models have implicated TRP channels in the development of hypertrophy. It has been shown that hypertrophic agonists upregulate the expression of TRPC1, TRPC3, and TRPC6 channels in cardiac myocytes, leading to activation of the Ca^2+^-(and ROS)–calmodulin–calcineurin-NFAT pathway which eventually results in cardiac hypertrophy. These hypertrophic changes are a pathological process basically involving inflammatory reactions [136, 189] and ultimately reduce the cardiac function increasing the mortality of experimental animals [118, 158–168].

More recent studies have added a new connection of TRP channels (TRPV2 and TRPM4) to cardiomyopathy. In the patients of cardiomyopathy as well as in its genetic and chemically induced animal models which typically show ventricular dilation, fibrosis, and severely compromised heart function, the expression of TRPV2 was found to be concentrated on the ventricular sarcolemma. Specific abrogation of TRPV2 activity by either overexpression of the amino-terminal domain of TRPV2 (amino acids 1–387) or treatment with chemical inhibitors ameliorated these pathological changes and the contractile function of the heart and improved the survival of the affected animals [171]. Since the excessive activity of TRPV2 was associated with increased production of ROS and phosphorylation of Ca^2+^/calmodulin-dependent protein kinase II (CaMK II), participation of inflammatory process is strongly suggested for these pathological changes. Thus, targeting TRPV2 may become a new clinical option to treat cardiomyopathy-associated heart failure.

In addition, a recent gene invalidation study showed that the activity of TRPM4 may be a prerequisite to preventing the development of eccentric ventricular hypertrophy of the heart. Indeed, TRPM4 has been shown to negatively regulate angiotensin II-induced cardiac hypertrophy owing its membrane depolarizing ability whereby the magnitude of store-operated Ca^2+^ entry which activates the calcineurin-NFAT hypertrophic pathway is negatively controlled [172, 173]. The correlation of these findings with inflammation is however unclear, since immunohistological examination revealed no obvious sign of fibrosis or hypertrophy but rather hyperplasia of smaller sized cardiomyocytes compared with normal ones.

### Myocardial infarction (MI)

In infarcted myocardium, necrotic cardiomyocytes release danger signals, activating an intense inflammatory response, by which complex cellular processes associated with injury, repair, and remodeling of the infarcted regions are activated [141]. In the MI period of animal models and human patients, the plasma level of brain-derived neurotrophic factor (BDNF) was found elevated along with tropomyosin-related kinase B (TrkB) and its downstream effector TRPC3 and TRPC6 channels. After BDNF treatment, the infarct size was markedly reduced and cardiac contractility was significantly restored, which seemed to be associated with decreased apoptotic response of cardiomyocytes. Since these beneficial effects of BDNF were reversed by pharmacological or functional inhibition of TRPC3/TRPC6 channels, these channels likely play a protective role against detrimental cardiac remodeling after MI [132, 174]. In a puzzling contrast, however, adenovirus-mediated overexpression of TRPC3/TRPC4/TRPC6 was reported to induce a hypertrophic response via the NFAT-mediated signaling in adult feline cardiomyocytes and accompany reduction of the contractility and catecholamine response due to increased spontaneous Ca^2+^ leak from the SR. In the same study, mice treated with MI procedures showed similarly enhanced TRPC1, TRPC3, TRPC4, and TRPC6 expression and Ca^2+^ channel activity along with induction of hypertrophic genes. These two lines of evidence have been interpreted to indicate the benefits of blocking the TRPC channels that improve post-MI structural remodeling and dysfunction. It is a matter of further investigation of what differences in experimental settings would make such disparate consequences of TRPC channel activation in the post-MI recovery.

In sharp contrast with a protective role of TRPM4 against dilated cardiomyopathy (see above), the deletion of the *trpm4* gene in mice rather improved survival and β-adrenergic cardiac reserve after experimentally induced ischemic heart failure [175, 185]. In addition, several lines of evidence support the ameliorative role of TRPV1 in myocardial infarction as found in atherosclerosis [176, 177, 179–182].

### Ischemia/reperfusion injury

Reperfusion of the ischemic myocardium is essential for rescuing it from the death. However, reperfusion itself causes additional myocardial injury termed “ischemia/reperfusion (I/R) injury” [190]. I/R injury in the heart occurs through innate immune responses involving TLR (TLR2, TLR4) and the Myd88- and Trif-dependent NF-κB-interferon-3 pathway, activation of which induces the release of proinflammatory and immunomodulatory cytokines [142]. Moreover, oxidative stress-induced acute inflammatory response is implicated in the development of I/R injury [191].

There are two conflicting reports linking TRPM2 channel to I/R injury. One study suggested that activation of neutrophil TRPM2 channel by ROS exacerbated myocardial I/R injury by upregulating the expression of endothelial adhesion molecules MAC-1 and LFA-1. This then resulted in a stronger adhesion of neutrophils on the coronary EC surface. Neutrophil accumulation in the myocardium is a key process that induces myocardial injury [191]. Thus, specific inhibition of neutrophil TRPM2 activity may serve as an effective means to mitigate the exacerbation of myocardial infarction. However, in a striking contrast to this view, an independent study proposed an opposing and more complex hypothesis. According to the findings of this study, genetic deletion of TRPM2 exacerbated the extent of myocardial dysfunction after I/R injury via increased generation (upregulation of NADPH oxidase) and decreased scavenging capacity (downregulation of superoxide anion disulmutases) of ROS. As pointed out by the authors themselves, the discrepancies between the above two studies may reflect small but essential differences in experimental designs, as well as the use of global knockout mice with different exon-targeting invalidation strategies [122]. In any case, it is almost undoubted that TRPM2 channel is among central players in immunoinflammatory reactions in the cardiovascular disease and thus a promising therapeutic target.

## Concluding remarks and perspectives

In this review, we summarized the current knowledge linking inflammation and TRP channels and attempted to provide new insights into the pathogenesis of various inflammatory CV diseases (refer to Table 1). In addition, we described the evidence that dysregulation or altered expression of TRP channels are in close association with inflammasome activation and the pathogenesis of CV diseases. Our major but tentative conclusions drawn from this review writing are as follows: TRPC1, TRPC3, TRPC6, and TRPM7 may be the most promising therapeutic targets in the subacute or chronic stages of some CV diseases (i.e., remodeling, proliferation), presumably via inhibition of persistent inflammasome activation as well as aberrant regulation of cytokine production and transcription factor activity. In case of ischemia/reperfusion, the molecules’ sensing/transducing oxidative stress appears contributory to the initiation of inflammation, wherein TRPM2 channel is the most plausible candidate (although roles of other oxidative stress-sensitive channels TRPV1, TRPM7, TRPA1, TRPC1, TRPC3, and TRPC6 should not be underestimated). Finally, dietary capsaicin, which appears to act as both sensory nerve (e.g., CLP sepsis model) and VSMC (e.g., atherosclerosis), TRPV1 activators, may have broad relevance to improving CV disorders through its anti-inflammatory actions.

The current research on the immunopathophysiology of TRP channels is still in its infant stage. It is thus strongly anticipated that further extensive investigations will greatly improve our yet premature understanding about it, which will pave a way to developing new effective therapies for inflammatory CV diseases.
